# Decreased level of RASSF6 in sporadic colorectal cancer and its anti-tumor effects both *in vitro* and *in vivo*

**DOI:** 10.18632/oncotarget.7852

**Published:** 2016-03-02

**Authors:** Erfei Chen, Fangfang Yang, Hongjuan He, Lei Lei, Ruitao Liu, Le Du, Jing Dong, Meng Wang, Jin Yang

**Affiliations:** ^1^ School of Life Sciences, Northwest University, Xi'an, P.R. China; ^2^ Institute of Preventive Genomic Medicine, Xi'an, P.R. China

**Keywords:** sporadic colorectal cancer, RASSF6, proliferation, apoptosis, loss-of-function mutation

## Abstract

Ras-association domain family protein 6 (RASSF6) is a member of tumor suppressor RASSFs family with a wide range of function from RAS interaction, Hippo signaling involvement to cell cycle and apoptosis regulation. RASSF6 is reported inactivated in various types of cancer. However, whether RASSF6 is associated with colorectal cancer and the underlying mechanisms have yet to be investigated. In our previous exome sequencing study, we found a somatic loss-of-function (LoF) mutation in *RASSF6* in one sporadic colorectal cancer (sCRC) patient, and two missense mutations in deep sequencing group of sCRC samples, implying the possibility that RASSF6 may be involved in the pathogenesis of sCRC. In this study, we demonstrate that RASSF6 acts as a tumor suppressor in colon cancer cells. Decreased level of RASSF6 was observed in adenocarcinoma compared to normal tissues, especially in advanced tumor cases. Further experiments showed exogenous introduction of RASSF6 into LoVo cells suppressed cell proliferation, migration, invasion, and induced apoptosis *in vitro* as well as tumor growth *in vivo*. In contrast, knockdown of RASSF6 in HT-29 cells showed the opposite effects. Taken together, our results suggest, in addition to epigenetics changes, functional somatic mutations may also contribute to the downregulation of RASSF6 and further participate in the pathogenesis of sCRC. RASSF6 may serve as a novel candidate against tumor growth for sCRC.

## INTRODUCTION

Colorectal cancer (CRC) is one of the most common cancer worldwide for both men and women, and the prevalence has been increasing over the past years. The occurrence of CRC is thought to be a joint effort of diet, lifestyle, environmental factors and acquired gene mutations [1]. However, for sCRC that accounts for about 80% of CRC, the spectrum of somatic mutations contributing to its pathogenesis is far from clear, in comparison to that of hereditary nonpolyposis colorectal cancer (HNPCC) [2]. The essential genetic alterations of sCRC include: *APC* gene mutations on the early development of adenomatous polyps; Activation of oncogene *KRAS* from early adenoma to late adenoma; Loss of tumor suppressor genes such as tumor protein p53 (*TP53*) in colorectal carcinoma [3]. Nevertheless, reported genetic markers can only explain little for the occurrence of sCRC.

Screening for new candidate genes through whole-genome has become a research hotspot in recent years. Next-Generation-Sequencing (NGS) is a powerful platform and potential for uncovering the genetic rules of cancer genome [4]. Growing evidence have confirmed that protein-coding exons accounts for only about 1% of the human genome, but covers most of functional variations (about 85%) associated with individual phenotypic. Therefore, exome sequencing can efficiently find mutations with low frequency and high penetrance [5]. Genome-wide identification of new candidate genes is critical for understanding the spectrum of sCRC genetic mutation and may throw a new light on gene therapy.

In our previous exome sequencing study, we focused on the LoF mutations that could impair protein expression in sCRC cancer genome, and identified a frameshift mutation of *RASSF6* in one sCRC patient. In addition with gene function analysis, we inferred *RASSF6* could be a new candidate gene for sCRC.

RASSF6 belongs to RASSFs family that involved in RAS interaction, Hippo signaling pathway and apoptosis induction [6, 7]. Many of RASSFs are frequently inactivated through loss of function mutations or promoter methylation [8]. It is reported downregulation of RASSF6 is involved in various solid cancer [9–12]. However, whether RASSF6 has an antitumor effect on colorectal cancer cells and the underlying regulation mechanisms remain unknown. In this study, to confirm our hypothesis that RASSF6 may play a role in the pathogenesis of sCRC, we performed comprehensive experiments both *in vitro* and *in vivo*. We investigated the somatic mutation status and RASSF6 expression level in sCRC samples, as well as the effects of RASSF6 on the biological behaviors of CRC cells. This article, as far as we know, is the first study concerning RASSF6 and sCRC.

## RESULTS

### Somatic mutations of *RASSF6* gene in sCRC patients

**Figure 1:**
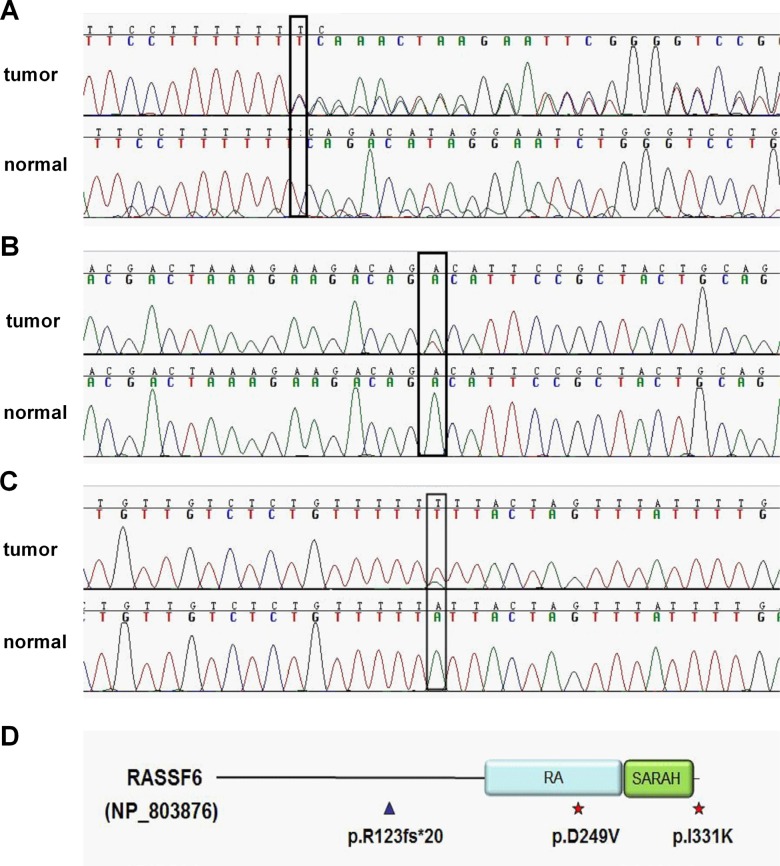
Somatic mutations detected in whole exome sequencing sample and deep sequencing samples (**A**) Resequencing of RASSF6 mutation in one of the whole exome sequencing samples. A heterozygous frameshift mutation (c.367insA (T), p.R123fs*20) in exon 5. (**B**) A heterozygous missense mutation (c.746A > T, p.D249V) in exon 9 identified in one of the deep sequencing samples. (**C**) A heterozygous missense mutation (c.992T > A, p.I331K) in exon 11. (**D**) Overview of 3 somatic mutations in protein level. RASSF6 contains two domains: RA domain and SARAH domain. △ represents frameshift mutation while ☆ represents missense mutation.

In our previous exome sequencing of 3 sCRC patients, we found somatic LoF mutation of *RASSF6* in one sample, an A-base insertion that cause frame-shift mutation (c.367insA) in exon 5 (NM_177532), leading to a truncated protein after encoding 20 amino acids (NP803876). This somatic mutation was not found in human cancers from the COSMIC project (mapped to GRCh38). For validation, we amplified the corresponding regions from the original sample (both tissue and normal blood control) using a PCR assay and carried out Sanger sequencing (Figure 1A).

To find similar functional mutations, we next carried out deep sequencing of RASSF6 coding region (exon 2- exon 11, NM_177532) in a group of 50 sCRC cases. Two missense mutations were detected and not yet reported in COSMIC (Figure 1B and 1C). The mutation impact was analyzed using the FATHMM-MKL algorithm. Where scores are ≥ 0.7 the mutation is classified as ‘pathogenic’, or ‘neutral’ if the core is ≤ 0.5 [13]. The score for D249V (c.746A > T) and I311K (c.992T > A) is 1.48 and 0.02, respectively. For D249V mutation, it affects a highly evolutionarily conserved RA domain of the gene (Figure 1D), with a polar aspartic acid replaced by hydrophobic nonpolar valine, and changing the chemical properties of the protein, likely representing a pathogenic mutation. For I311K mutation, this residue locates at the end of RASSF6 coding region, as a consequence, the effect is limited.

### RASSF6 expression in sCRC samples and CRC cell lines

**Figure 2:**
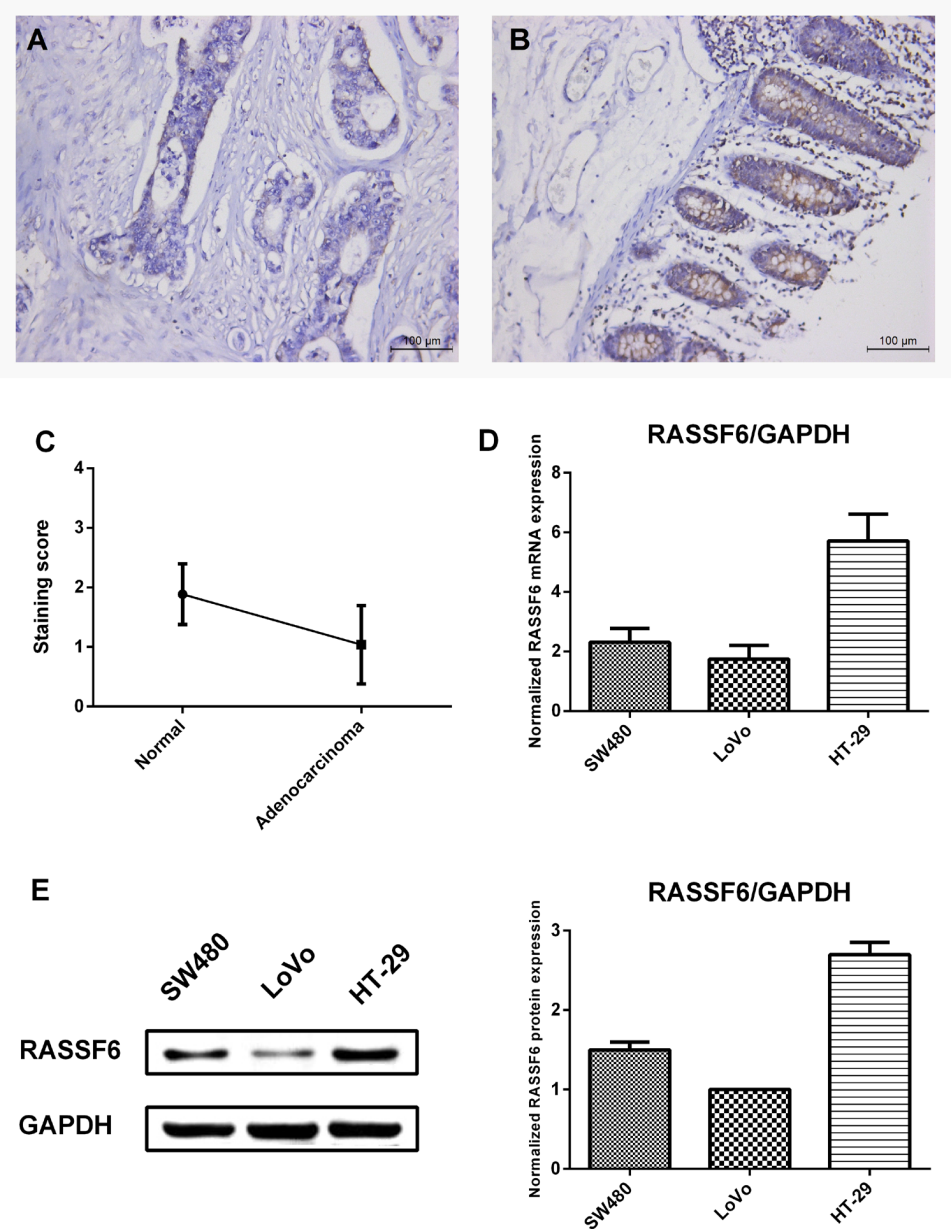
RASSF6 expression in colon tissues and CRC cell lines (**A**) Immunohistochemical staining of RASSF6 expression in tissue microarray. Weak or negative staining for RASSF6 was detected in adenocarcinoma. (**B**) Adjacent benign glands were showing strong RASSF6 staining. (A and B) are representative images from the same patient. (**C**) Comparison of staining score between 52 cases of adenocarcinoma and adjacent normal tissue. Staining score is classified as weak or negative (0), mild (1), moderate (2), and strong (3) (***P < 0.001, paired *t* test). (**D**) Transcriptional level of RASSF6 in colorectal cancer cell lines (SW480, LoVo, and HT-29) was analyzed using quantitative real time-PCR. RASSF6 was expressed in all cell lines, relatively high in HT-29 cells and low in LoVo cells, with GAPDH as the normalized reference gene. (**E**) Western blot analysis of RASSF6 protein levels that normalized to GAPDH expression. The result is consistent with transcriptional level analysis. All data are shown as mean ± SE.

No reports concerning RASSF6 expression in CRC were reported yet. To verify the expression level of RASSF6 in CRC, we carried out immunohistochemistry in a panel of tissue array containing 52 samples of colorectal adenocarcinoma and adjacent normal colon tissue (Supplementary Table S1). RASSF6 expression was observed in 80.8% (42/52) of the adenocarcinoma cases and 100% (52/52) of the normal cases (Table 1). RASSF6 was highly expressed in benign glands compared with adenocarcinoma (Figure 2A–2C), and no strong RASSF6 staining was found in adenocarcinoma cases. 67.4% (35/52) samples detected lower expression of RASSF6 in adenocarcinoma than adjacent normal tissue, 28.9% (15/52) samples showed no difference and only 3.8% (2/52) samples showed higher expression in adenocarcinoma (Supplementary Table S1). Based on clinical stage, we classified sCRC patients into two subgroups, with stage I as group 1, stage II and stage III as group 2. Notably, RASSF6 was significantly downregulated in group 2 compared with group 1 (39.1% versus 3.4%, *P* < 0.01, negative or weak expression ratio) (Table 2). This result may indicate that decreased level of RASSF6 may correlate with patients of advanced tumor stage. No significance was observed between RASSF6 expression and age, sex of 52 colorectal adenocarcinoma.

**Table 1:** RASSF6 staining and scoring in human tissue array

	Total no. of samples	No. of RASSF6 positive samples	%positive (No./total)	*P* value
Normal colorectal tissue	52	52	100	0.0012*
Adenocarcinoma	52	42	80.8	

* Statistical difference in the percentage of RASSF6 positive samples between sporadic colorectal adenocarcinoma and adjacent normal tissues.

For the expression analysis in cell lines, we aimed to assess which cell line could be the model for our gain-of-function and loss-of function analysis and not to indicate any difference in expression levels. The transcriptional and protein level of RASSF6 in a panel of CRC cell lines (HT-29, SW480, and LoVo) was first analyzed by way of quantitative real time-PCR. RASSF6 mRNA was expressed in all 3 CRC cell lines, with relative higher expression in HT-29 and lower expression in LoVo cells (Figure 2D). In accordance with quantitative real-time PCR analysis, we found similar protein expression level trend (Figure 2E). Interestingly, LoVo cells are isolated from left supraclavicular region with high metastatic ability as compared to low metastatic ability cell lines HT-29 and SW480. Together with these findings, we hypothesize silencing or downregulation of RASSF6 may be related to the degree of cell differentiation or malignancy. Consequently, we chose LoVo cells as a gain-of-function model and HT-29 cells as a loss-of-function model to investigate the effect of RASSF6 on cell biological behaviors.

### RASSF6 inhibits cell proliferation and induces apoptosis of CRC cells

**Figure 3:**
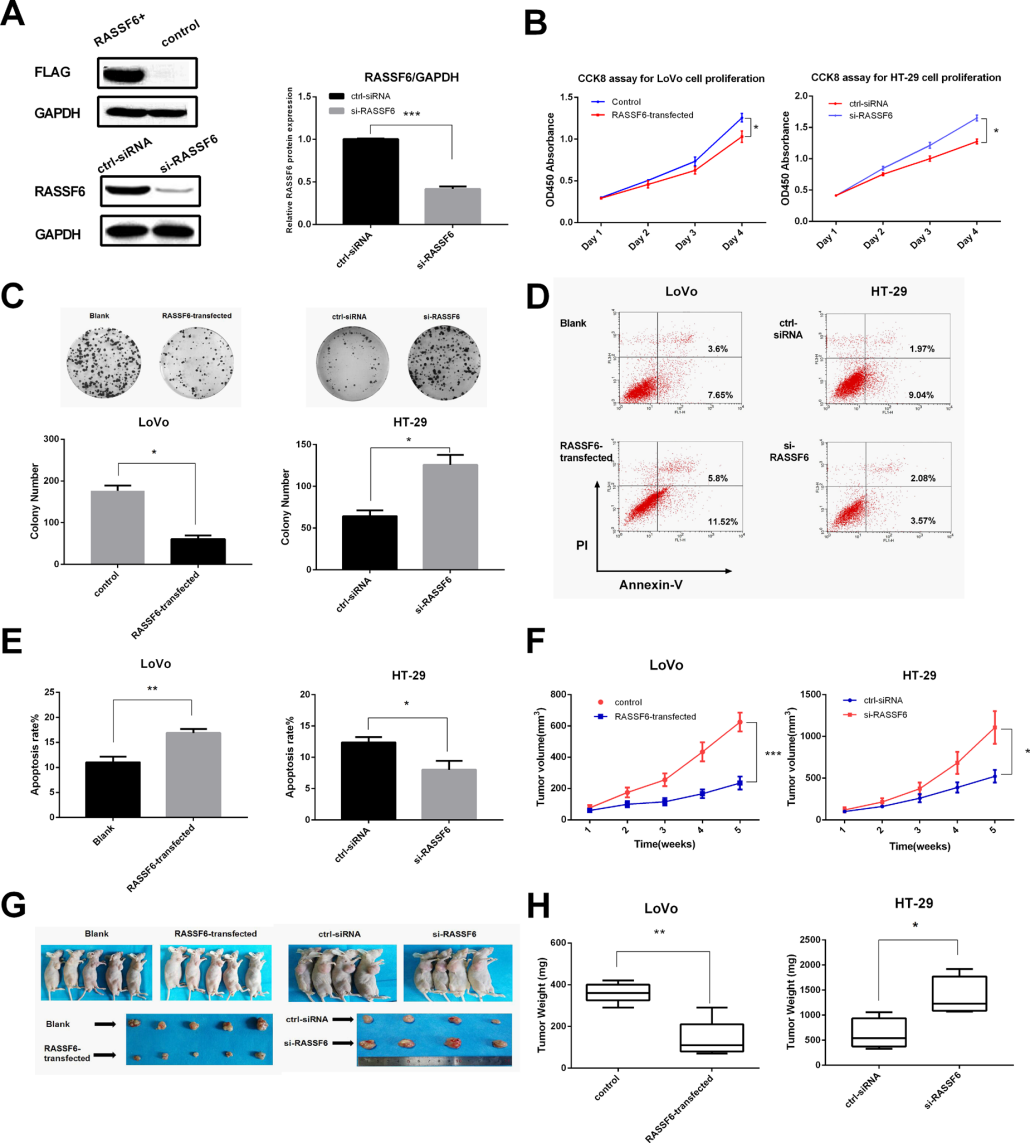
RASSF6 inhibits cancer cell proliferation and induces apoptosis (**A**) The overexpression and knockdown efficiency were confirmed by western blot, with anti-flag antibody and specific anti-RASSF6 antibody used respectively. (**B**) LoVo and HT-29 cell proliferation was determined by CCK8 assays after transfection, on day 1, 2, 3, 4. (**C**) The effects of RASSF6 on the colony forming ability of LoVo and HT-29 cells. Images are representative of three independent experiments. (**D** and **E**) The effects of RASSF6 on LoVo and HT-29 cells apoptosis was detected by flow cytometry. (**F**) Tumor volume was assessed once a week with a total period of 5 weeks (*n =* 5 or 4). (**G**) Images of nude mice bearing the tumors and isolated tumors from injected mice. (**H**) Weights of the isolated tumor (*n =* 5 or 4). All data are showed as mean ± SE. *P < 0.05, **P < 0.01, ***P < 0.001.

To assess the role of RASSF6 in the growth of CRC, cell proliferation was measured using CCK8 assay, and we observed that RASSF6-transfected LoVo cells showed a decreased growth rate over time than control (empty vector-transfected cells), whereas knockdown of RASSF6 in HT-29 cells promoted cell proliferation (*P < 0.05, Figure 3B). Colony formation assay further showed that overexpression of RASSF6 resulted in tumor growth inhibition (*P < 0.05, Figure 3C).

Cell apoptosis was measured 48 h after transfection of RASSF6 expression vector or RASSF6 siRNA by flow cytometry. The percentage of apoptotic cells in RASSF6-transfected group was higher than control group (**P < 0.01, Figure 3D). Especially, knockdown of RASSF6 significantly decreased early apoptotic cells. These results confirmed that RASSF6 has a potent effect on apoptosis induction of CRC cells.

To further examine RASSF6 function *in vivo*, we engineered LoVo cells to stably express RASSF6 or HT-29 cells treated with RASSF6 inhibitor, and BALB/c nude mice were applied to evaluate the effect of RASSF6 on tumorigenicity (Figure 3F). Compared with the control group, RASSF6-stably expressed cells revealed a decreased tumor size and tumor weight, whereas low RASSF6 level in HT-29 promoted tumor growth as compared to control siRNA group (Figure 3G and 3H).

### RASSF6 inhibits the migration and invasion ability of CRC cells

**Figure 4:**
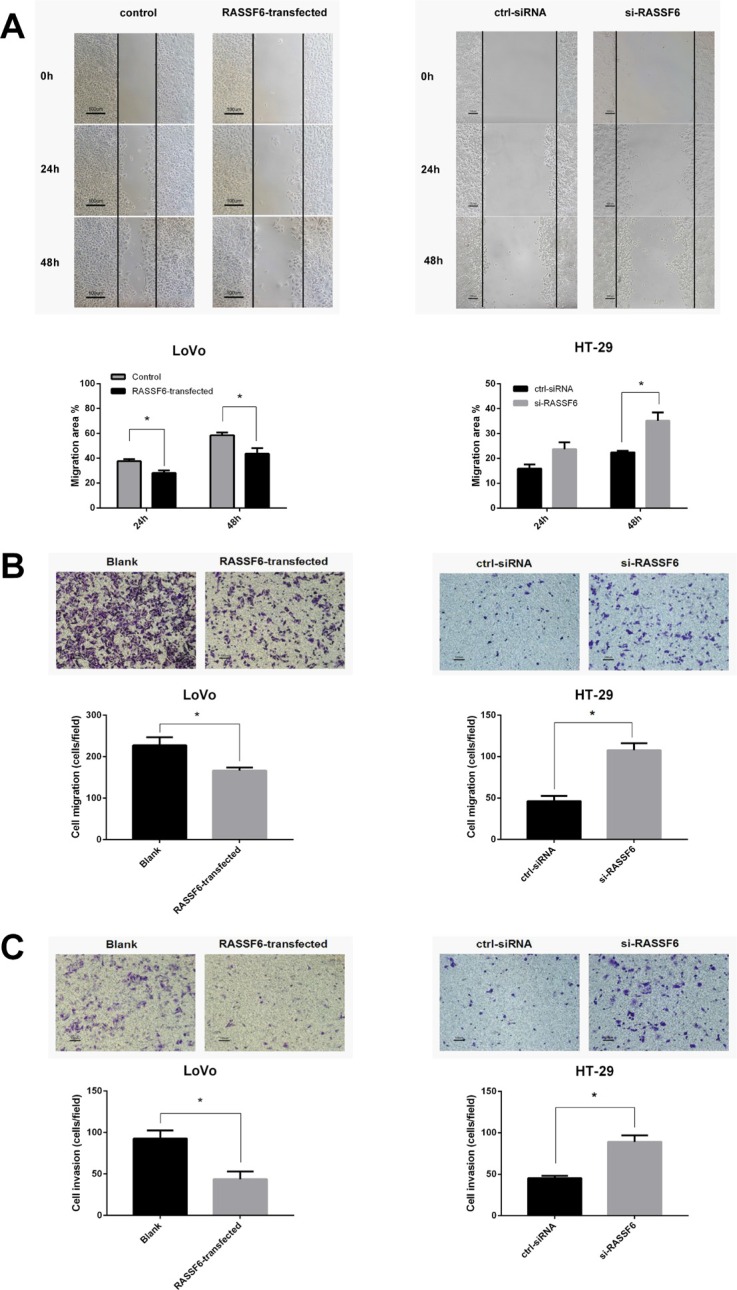
RASSF6 inhibits the migration and invasion ability of colorectal cancer cells (**A**) Wound scratch assays were performed in LoVo cells with transfection of empty vector as control or RASSF6 (Left, A), and HT-29 cells with transfection of scrambled control siRNA or RASSF6 siRNA respectively. Images were visualized at 0 h, 24 h, and 48 h at a magnification of 100 ×. RASSF6 highly-expressed cells showed lower migration ability than RASSF6-low cells. (**B**) The migration ability was also assessed using Transwell chamber without Matrigel (18 h for LoVo and 48 h for HT-29 cells). (**C**) The effects of RASSF6 on the invasion ability of cancer cells were assessed by Matrigel-invasion assay (24 h for LoVo and 48 h for HT-29 cells). Representative images were visualized at a magnification of 100 ×. All data are showed as mean ± SE of three independent experiments, and for every single experiment, the data are mean of at least three fields, *P < 0.05.

Wound scratch assays and Transwell chamber (Matrigel-free) were performed to check the effect of RASSF6 on cancer cell migration ability. As shown in Figure 4A and 4B, the LoVo cell migration ability was impaired by RASSF6 compared to controls. Conversely, Knockdown of RASSF6 in HT-29 cells rescued the migration ability. Additionally, we explored the effect of RASSF6 on the invasion ability of cancer cells using a Matrigel invasion assay. Consistently, a significantly decreased invasion was observed in RASSF6 high expressing cells (*P < 0.05, Figure 4C). These findings suggest that RASSF6 regulates the migration and invasion capacities of CRC cells *in vitro*.

## DISCUSSION

The biomarker of sCRC is far from clear and identified, whole exome sequencing based on LoF mutation screening is a powerful method for novel candidate genes identification of sporadic cancer. In this study, we are here for the first time to show RASSF6 screened by whole exome sequencing may serve as a novel candidate against tumor for sCRC. Previous reports have shown that, similar to other members of RASSF family, RASSF6 acts as a tumor suppressor. Downregulation of RASSF6 is involved in neuroblastoma, melanoma, nasopharyngeal cancer and childhood acute lymphocytic leukemia, and its decreased expression level is also associated with poor survival of pancreatic ductal adenocarcinoma and gastric cancer [9–12, 14, 15]. However, to date, the expression analysis of RASSF6 and its underlying mechanisms of action in sCRC have not been comprehensively investigated. Intriguingly, in our previous whole exome sequencing study, we performed LoF mutations screening strategy and found somatic mutation of *RASSF6* in one sCRC patient. In this view, we hypothesized functional mutations may also contribute to the loss of RASSF6 in addition to promoter methylation.

We first collected 50 sCRC samples for deep sequencing of *RASSF6* coding regions and found two novel missense mutations, c.746A > T and c.992T > A. The frameshift mutation (c.367insA) and c.764A > T may be pathogenic. The frameshift mutation leads to the truncated protein without encoding RA and SARAH domains. The missense mutation causes the amino acid change from aspartic acid to valine which may impair the structure of RA domain.

Next, we carried out immunohistochemistry staining of tissue array and found significant downregulation of RASSF6 in sCRC samples, 67.4% (35/52) showed decreased level of RASSF6 as compared with self-adjacent normal tissue. High frequency of downregulation of RASSF6 in sCRC samples showed RASSF6 may serve as a critical gene and biomarker in the pathogenesis of sCRC. Specifically, a significant difference in RASSF6 expression was observed between late clinical stage patients and early stage sCRC patients (Table 2). Moreover, in high metastatic ability LoVo cells, the RASSF6 levels were downregulated as compared with HT-29 cells. Collectively, our expression results showed decreased level of RASSF6 might be associated with aggressive CRC phenotypes (high metastatic ability cell lines and advanced tumor patients). The downregulation or silence of tumor suppressor RASSF6 in the late stage of sCRC could aggravate the progress of malignancy.

**Table 2:** Correlation of RASSF6 expression to age, sex and clinical stage of 52 colorectal adenocarcinoma

Variables	Cases	RASSF6 expression			χ^2^	*P* Value
		0	1+	2+		
Age (years)						
> 60	24	6	12	6	1.3	0.522
≤ 60	28	4	18	6		
Sex						
Male	33	5	22	6	2.98	0.2254
Female	19	5	8	6		
Clinical stage						
I	29	1	19	9	10.99	0.0041*
II–III	23	9	11	3		

RASSF6 expression was classified as 0: weak or negative staining, 1+: mild positive, 2+ moderate positive. Clinical stage was classified into two subgroups, with stage I as group 1, stage II and stage III as group 2.

* Statistical significance in RASSF6 between group 1 and group 2.

To further confirm our hypothesis that *RASSF6* acts as a tumor suppressor, and loss of RASSF6 might be involved in the pathogenesis of sCRC, we performed systematic experiments both *in vitro* and *in vivo*. Overexpression of RASSF6 can inhibit cell proliferation, migration, invasion, and induce apoptosis of LoVo cells. In contrast, knockdown of RASSF6 in HT-29 cells induced opposite effects.

To fully unravel the roles RASSF6 plays in tumorigenesis of sCRC, further experiments based on our research are still needed to clarify the underlying mechanism. In further studies, we would like to collect a larger set of sCRC samples for deep sequencing, and if possible, to analyze the promoter methylation of *RASSF6*. Hypermethylation status of *RASSF6* were found in metastatic melanoma, neuroblastoma and across the whole CpG island in leukemia cell lines [9, 11, 14]. Is it a common event in various solid cancer? Or LoF mutations from DNA level contribute to the silence of RASSF6. In addition, it will be of necessity to enlarge samples to clarify the expression conditions in sCRC.

It will be especially interesting to dissect the mechanism RASSF6 plays in the colon cancer model and its interaction with other molecules in signaling pathways. RASSF6 was first characterized and identified as a novel member of RASSF family in 2007 [16]. Structurally, RASSF6 contains the Ras-association domain and protein interaction domains like SARAH that can bind the pro-apoptotic kinases MST1 and MST2 [17, 18]. Previous research on the mechanism of RASSF6 mainly focuses on how it works on apoptosis. RASSF6 can trigger both apoptosis caspase-dependent or caspase-independent pathways thus to induce apoptosis. The study found enhanced expression of RASSF6 induced apoptosis via Bax activation and cytochrome c release induction [19]. Furthermore, RASSF6 is also involved in signaling transduction. It exhibits a dual role in Hippo pathway, as an inhibitor that regulates cell proliferation or mediates apoptosis independently of the canonical Hippo pathway [20]. In addition, as a common function of C-terminal RASSF members, RASSF6 can bind to MDM2 and facilitate its protein degradation and stabilize p53, thereby regulating apoptosis and cell cycle [21].

In conclusion, our results confirmed *RASSF6*, as an important tumor suppressor gene, plays a critical role in the pathogenesis of sCRC, and its decreased level could be a biomarker for warning and prevention of sCRC.

## MATERIALS AND METHODS

### Sample collection and deep sequencing of RASSF6 coding region

All of the patients provided informed consent for collection of tissues. All samples (tissue array and 3 original samples for whole exome sequencing and 50 sCRCs for deep sequencing) are confirmed with no family history of colorectal cancer.

For 3 cases in whole exome sequencing, we found no reported mutation for hereditary CRC of Chinese Han population (Supplementary Table S2). For 50 cases in deep sequencing, tumor DNA and matched normal tissue DNA were extracted using QIAamp DNA FFPE Tissue Kit (QIAGEN) according to the manuals. Sanger sequencing was carried out firstly in tumor DNA, the coding region of RASSF6 (exon 2–10, NM_177532) (Primers for each exon see were shown in Supplementary Table S3) was amplified and sequenced. Mutations found in tumor sample were resequenced in matched normal tissue DNA to exclude the possibility of single nucleotide polymorphism (SNP). Somatic mutations were visualized and analyzed by Sequencher 5.1.

### Cell culture

Human colorectal adenocarcinoma cells (HT-29, LoVo, and SW480) were purchased from ATCC (American Type Culture Collection) and cultured in complete medium (with 1% penicillin-streptomycin (HyClone, Logan, Utah, USA)) recommended by ATCC. Cells resuspended in complete medium free of penicillin-streptomycin are used for further experiments after detaching with trypsin (Corning, MA, USA).

### Construction of expression vectors and transfection

The full-length cDNA of RASSF6 was obtained from 293T cDNA by PCR using the following primers: sense 5′-CGG GAT CCA TGA CTA TGA TGG CTC ACC AG-3′ / antisense: 5′-GCG TCG ACC TAA ACT GTT GTC TCT GTT T-3′. The obtained fragment was then inserted into the BamHI/SalI sites of pEF-BOS-EX, a powerful mammalian expression vector donated by J. Jiang of Hokkaido University. Transfection was carried out using X-tremeGENE HP DNA Transfection Reagent (Roche Applied Sciences). 90% transfection efficiency was ensured using a fluorescence expression vector with eGFP (pIRES2).

### RNA interference

RASSF6 siRNA and negative control siRNA were purchased from GenePharma (Shanghai, China). HT-29 cells were transfected using HiPerFect transfection reagent (Qiagen, Valencia, CA, USA) at a confluency of 30–40%. The transfection efficiency was detected using 5′-FAM control siRNA, and the knockdown efficiency was verified by qRT-PCR and western blot. The optimum siRNA for RASSF6 (5′- GAC CCA GAU UCC UAU GUC UdTdT-3′/5′-AGA CAU AGG AAU CUG GGU CdTdT-3′) was selected for experiments. For *in vivo* tumor growth assay, siRNA was modified by 2′-Ome.

### RNA extraction and quantitative real-time PCR

Total RNA Miniprep Kit (Sigma, St Louis, MO) was used to extract RNA from cell lines according to the manuals. The isolated RNA was then reverse transcribed to cDNA (Takara, Japan). Quantitative real-time PCR amplifications were performed using the SYBR Premix Ex Taq^™^ II kit (TAKARA, Japan) by CFX96^™^ real-time PCR detection system. Quantification was adjusted using the house-keeping genes GAPDH. A total volume of 25 μl with the condition (95°C, 30s for initial denaturation, 40 cycles of 95°C for 5s and 60°C for 30s in addition with a Melt curve reaction) was performed. Each reaction was performed in triplicate, and the mRNA level of RASSF6 for cancer cell lines were calculated according to the formulas 2^−ΔCt^.

### Western blot analysis

Total protein was lysed in RIPA buffer (protease inhibitor added). A BCA assay kit (Pierce, Rockford, IL) was used to quantify protein concentration. Equal amounts of protein (20 or 30 μg) were separated on a 12% SDS–PAGE gel and then electroblotted onto nitrocellulose membranes. After blocking with 5% nonfat-dried milk in tris-buffered saline with 0.05% Tween20 (TBST) buffer for 1 h at room temperature, the membranes were incubated overnight at 4°C with rabbit anti-RASSF6 (1:200, Sigma, St Louis, MO) or mouse anti-GAPDH (1:2000, Bioworld, Beijing, China). The membranes were incubated with corresponding IgG-HRP secondary antibody (1:5000, Bioworld, Beijing, China) after 3 washes with TBST. The bands were visualized with HRP Substrate (Millipore Corporation, USA) and then exposed to KODAK film. Quantification was carried out using the imaging program Image J (NIH) and the value was then normalized to corresponding GAPDH control.

### Immunohistochemistry staining

A tissue microarray was used in this study. Immunohistochemistry staining was performed, as described previously [22]. For antigen retrieval, tissue sections were boiled in 0.01 M citrate solution (pH 6.0) and incubated with primary antibody to RASSF6 (1:150, Sanying, Wuhan, China). Tissue sections were observed with a standard light microscope (Leica).

### Cell proliferation assay

For proliferation studies, Cell Counting Kit-8 (Dojindo Laboratories, Japan) was used according to the manufacturer's instructions. After 24 hours of transfection, cells were inoculated at a density of 2,000 per well into a 96-well plate with 6 replicates. To measure the proliferative activity, 10 μl CCK-8 solution plus 90 μl complete medium were added into each well After incubation at 37°C for 3 hours, the number of viable cells was measured at a wavelength of 450 nm (a continuous detection of 4 days).

### Colony formation assay

500 cells were seeded in 6-well plates. The cells were cultured for approximately 14 days, and the medium was changed every 2–3 days. At indicated time point, cells were washed twice with PBS, then fixed with 4% paraformaldehyde, and stained with 0.1% crystal violet. The total number of colonies (> 50 cells) was counted.

### Apoptosis detection assay

Cell apoptosis was determined by Annexin V/PI apoptosis detection kit (KeyGEN, Nanjing, China) following the protocol and the method was performed as described previously [23]. Early and late apoptotic cells were analyzed by FACSCalibur flow cytometer (BD Bioscience, USA).

### *In vitro* migration and invasion assays

To check the ability of cell migration, we performed wound healing assay. LoVo cells were cultured in a 6-well plate, and 48 hours after transfection, the cell layer was then carefully scratched with a 10 μl pipet tip. After washing off floating cells with PBS, LoVo cells were maintained in Opti-MEM medium (Corning, MA, USA). Cell migration into the wounded area was observed and photographed under a microscope (Nikon Instruments Inc.) at a magnification of 100 × (0, 24 h, and 48 h). 3–5 fields were randomly picked to calculate healing percentage and the experiments were performed in triplicate.

Cell invasion ability was evaluated using Transwell chambers (Corning, MA, USA) coated with Matrigel (BD Biosciences, Bedford, MA, USA). 5 × 10^4^ LoVo cells or 10^5^ HT-29 cells were suspended in 100 μl serum-free medium and then plated in the upper chamber of the 24-Well, with 600 μl medium containing 10% FBS in the lower chamber. The cells were incubated at 37°C for 24 hours (LoVo cells) or 48 hours (HT-29 cells). The noninvasive cells in the upper chambers were removed with cotton swabs. The invaded cells on the lower chamber were fixed in 4% paraformaldehyde solution for 30 minutes and then stained with 0.1% crystal violet. The cells were counted under a microscope at a magnification of 100 ×. At least 6 fields were randomly picked and three independent experiments were conducted.

### Xenograft model in nude mice

Male balb/c nude mice (3–5 weeks old) were purchased from Animal Center at Medical College, Xi'an Jiaotong University. All mice were housed and maintained under specific pathogen-free conditions and all experiments were carried out according to the ethical guidelines established by Animal Care and Use Committee of Xi'an Jiaotong University. 2 × 10^6^ cells (treated LoVo cells or untreated HT-29 cells) were resuspended in 200 μl serum-free medium and then injected to the subcutaneous of the right axilla of nude mice (*n =* 4 or 5 mice/group). For knockdown trial, when the tumor volume reached about 80–100 mm^3^, the nude mice were divided into two groups, RASSF6 siRNA or control siRNA diluted in saline was injected into the tumor at a dose of 30 μg every week. The tumors' dimensions were monitored with vernier calipers for a total period of 5 weeks, and the tumor volume was calculated using the following formula: 0.5 × a × b^2^, where a represents the longer diameter and b represents the corresponding perpendicular shorter diameter.

### Statistical analysis

Data generated from three independent experiments were presented as the mean ± SE. GraphPad Prism (GraphPad Software, Inc.) was used for unpaired *t*-test, paired *t*-test, and Chi-square test. *P* value < 0.05 was considered statistically significant.

## SUPPLEMENTARY MATERIALS FIGURE AND TABLES



## References

[1] Slattery ML (2000). Diet, lifestyle, and colon cancer. Semin Gastrointest Dis.

[2] Mundade R, Imperiale TF, Prabhu L, Loehrer PJ, Lu T (2014). Genetic pathways, prevention, and treatment of sporadic colorectal cancer. Oncoscience.

[3] Gonzalez-Gonzalez M, Garcia JG, Montero JA, Fernandez LM, Bengoechea O, Munez OB, Orfao A, Sayagues JM, Fuentes M (2013). Genomics and proteomics approaches for biomarker discovery in sporadic colorectal cancer with metastasis. Cancer Genomics Proteomics.

[4] Mardis ER, Ding L, Dooling DJ, Larson DE, McLellan MD, Chen K, Koboldt DC, Fulton RS, Delehaunty KD, McGrath SD, Fulton LA, Locke DP, Magrini VJ (2009). Recurring mutations found by sequencing an acute myeloid leukemia genome. N Engl J Med.

[5] Meyerson M, Gabriel S, Getz G (2010). Advances in understanding cancer genomes through second-generation sequencing. Nature Reviews Genetics.

[6] Richter AM, Pfeifer GP, Dammann RH (2009). The RASSF proteins in cancer; from epigenetic silencing to functional characterization. Biochim Biophys Acta.

[7] Sherwood V, Recino A, Jeffries A, Ward A, Chalmers AD (2010). The N-terminal RASSF family: a new group of Ras-association-domain-containing proteins, with emerging links to cancer formation. Biochem J.

[8] Volodko N, Gordon M, Salla M, Ghazaleh HA, Baksh S (2014). RASSF tumor suppressor gene family: biological functions and regulation. FEBS Lett.

[9] Djos A, Martinsson T, Kogner P, Caren H (2012). The RASSF gene family members RASSF5, RASSF6 and RASSF7 show frequent DNA methylation in neuroblastoma. Mol Cancer.

[10] Huang Z, Li W, Lin S, Fang X, Zhang C, Liao Z (2014). Identification of novel tumor suppressor genes down-regulated in recurrent nasopharyngeal cancer by DNA microarray. Indian J Otolaryngol Head Neck Surg.

[11] Mezzanotte JJ, Hill V, Schmidt ML, Shinawi T, Tommasi S, Krex D, Schackert G, Pfeifer GP, Latif F, Clark GJ (2014). RASSF6 exhibits promoter hypermethylation in metastatic melanoma and inhibits invasion in melanoma cells. Epigenetics.

[12] Wen Y, Wang Q, Zhou C, Yan D, Qiu G, Yang C, Tang H, Peng Z (2011). Decreased expression of RASSF6 is a novel independent prognostic marker of a worse outcome in gastric cancer patients after curative surgery. Ann Surg Oncol.

[13] Shihab HA, Gough J, Cooper DN, Day IN, Gaunt TR (2013). Predicting the functional consequences of cancer-associated amino acid substitutions. Bioinformatics.

[14] Hesson LB, Dunwell TL, Cooper WN, Catchpoole D, Brini AT, Chiaramonte R, Griffiths M, Chalmers AD, Maher ER, Latif F (2009). The novel RASSF6 and RASSF10 candidate tumour suppressor genes are frequently epigenetically inactivated in childhood leukaemias. Mol Cancer.

[15] Ye HL, Li DD, Lin Q, Zhou Y, Zhou QB, Zeng B, Fu ZQ, Gao WC, Liu YM, Chen RW, Li ZH, Chen RF (2015). Low RASSF6 expression in pancreatic ductal adenocarcinoma is associated with poor survival. World J Gastroenterol.

[16] Allen NP, Donninger H, Vos MD, Eckfeld K, Hesson L, Gordon L, Birrer MJ, Latif F, Clark GJ (2007). RASSF6 is a novel member of the RASSF family of tumor suppressors. Oncogene.

[17] van der Weyden L, Adams DJ (2007). The Ras-association domain family (RASSF) members and their role in human tumourigenesis. Biochim Biophys Acta.

[18] Gordon M, Baksh S (2011). RASSF1A: Not a prototypical Ras effector. Small GTPases.

[19] Ikeda M, Hirabayashi S, Fujiwara N, Mori H, Kawata A, Iida J, Bao Y, Sato Y, Iida T, Sugimura H, Hata Y (2007). Ras-association domain family protein 6 induces apoptosis via both caspase-dependent and caspase-independent pathways. Exp Cell Res.

[20] Ikeda M, Kawata A, Nishikawa M, Tateishi Y, Yamaguchi M, Nakagawa K, Hirabayashi S, Bao Y, Hidaka S, Hirata Y, Hata Y (2009). Hippo pathway-dependent and -independent roles of RASSF6. Sci Signal.

[21] Iwasa H, Kudo T, Maimaiti S, Ikeda M, Maruyama J, Nakagawa K, Hata Y (2013). The RASSF6 tumor suppressor protein regulates apoptosis and the cell cycle via MDM2 protein and p53 protein. J Biol Chem.

[22] Liu W, Lu M, Liu B, Huang Y, Wang K (2012). Inhibition of Ca (2+)-activated Cl (−) channel ANO1/TMEM16A expression suppresses tumor growth and invasiveness in human prostate carcinoma. Cancer Lett.

[23] Ma J, Duan W, Han S, Lei J, Xu Q, Chen X, Jiang Z, Nan L, Li J, Chen K, Han L, Wang Z, Li X (2015). Ginkgolic acid suppresses the development of pancreatic cancer by inhibiting pathways driving lipogenesis. Oncotarget.

