# Supplementary material for: Decreased level of RASSF6 in sporadic colorectal cancer and its anti-tumor effects both in vitro and in vivo
Source: Oncotarget. 2016 Mar 2;7(15):19813–23. doi: 10.18632/oncotarget.7852 (PMC4991420; doi:10.18632/oncotarget.7852)
Supplement: Supplementary file 1 [file oncotarget-07-19813-s001.pdf]

Decreased level of RASSF6 in sporadic colorectal cancer and its anti-tumor effects both *in vitro* and *in vivo*

Supplementary Materials

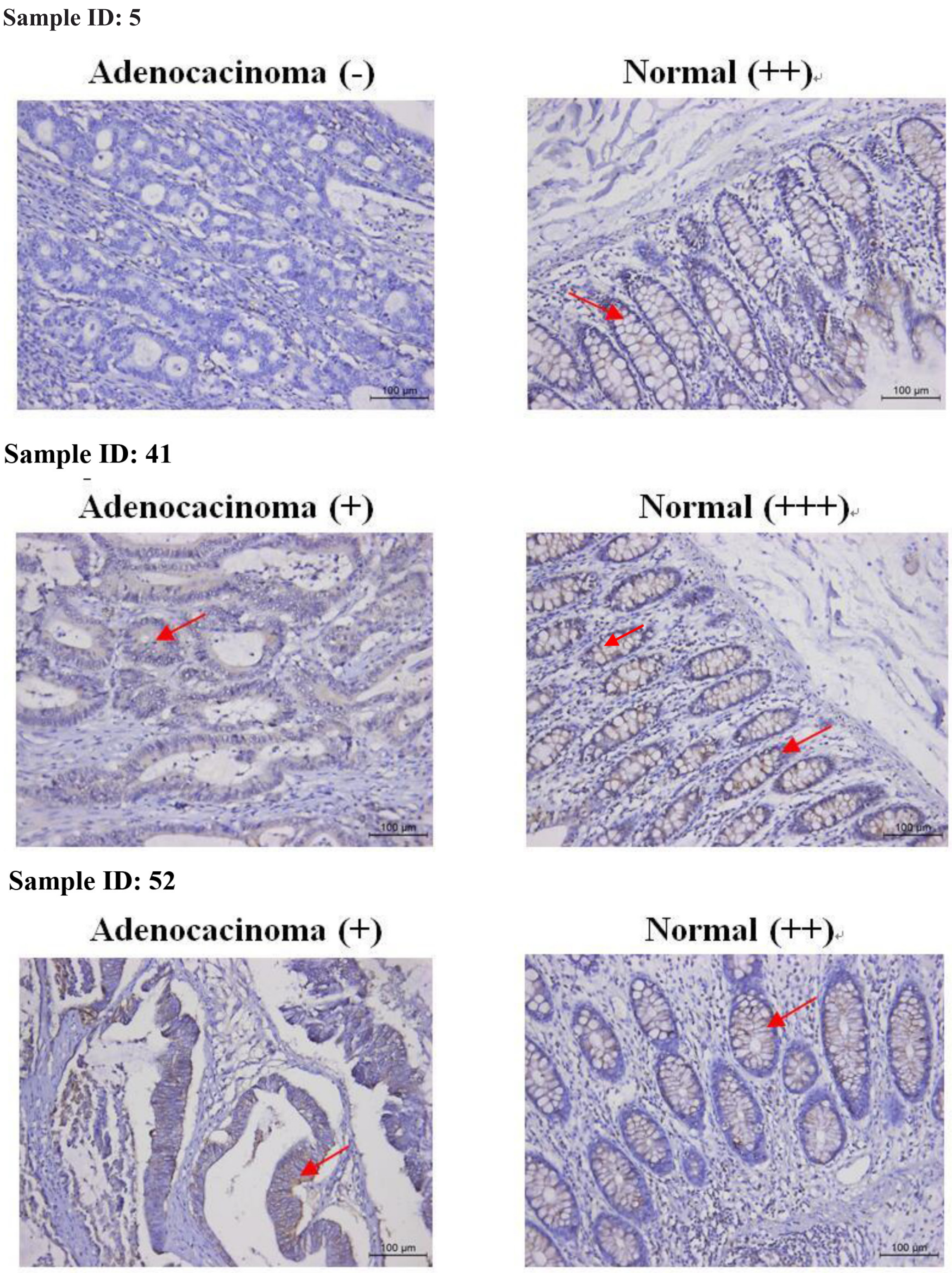

Supplementary Figure S1: Representative images of immunohistochemistry staining.

Supplementary Table S1: Clinical data of 52 sCRC patients in tissue array

Sample ID	Age (years)	Gender	Stage at surgery	RASSF6 staining score	
				Adenocarcinoma	Adjacent normal tissue
1	52	F	II	+	++
2	48	M	II	–	++
3	32	M	II	++	++
4	56	F	II	–	++
5	73	F	III	–	++
6	45	M	II	+	++
7	48	M	I	+	+
8	54	F	I	++	++
9	71	M	I	+	++
10	70	M	I	+	++
11	66	F	I	+	+
12	79	M	II	+	+++
13	35	M	III	+	++
14	67	F	III	+	++
15	65	M	II	–	+++
16	75	M	II	–	+
17	74	M	II	+	++
18	34	M	I	+	+
19	62	M	I	–	++
20	56	M	I	++	+
21	85	F	I	++	++
22	61	M	I	+	+++
23	44	M	I	+	++
24	55	M	I	+	+
25	57	F	I	+	++
26	63	M	I	+	++
27	60	M	II	+	++
28	57	M	II	+	++
29	72	M	I	+	++
30	51	F	I	++	+
31	52	M	I	++	++
32	71	M	I	++	++
33	61	F	I	++	++
34	51	M	I	+	+
35	62	M	I	++	++
36	64	F	I	+	++

37	61	F	I	+	++
38	68	F	I	++	++
39	57	F	II	–	++
40	74	M	II	++	++
41	70	M	II	+	+++
42	68	F	II	–	+
43	28	F	II	++	++
44	55	M	II	+	++
45	61	F	II	–	++
46	41	M	III	+	++
47	51	M	II	–	+
48	32	M	I	+	++
49	58	F	I	+	++
50	59	M	I	+	++
51	58	M	I	+	++
52	38	F	I	+	++

Supplementary Table S2: Diagnostic mutation for hereditary CRC of Chinese Han population

Gene	Exon	Allele change	Effect
MLH1	3	c.298C > T	Arg > Stop
	3	c.229T > C	Cys > Arg
	3	c.250A > G	Lys > Glu
	6	c.545 + 3A > G	–
	6	503_504insA	Framshift
	6	c.472delA	Frameshift
	9	c.790 + 1G > A	Splice_site
	16	c.1761_1762insG	Frameshift
	16	c.1771delG	Frameshift
	16	c.1846_1848delAAG	Del Lys
	19	c.2172_2173insG	Frameshift
	19	c.2263A > G	Frameshift
MSH2	7	c.1216_1219dupCGAC	Frameshift
	12	c.1808A > G	Asp > Gly

Supplementary Table S3: Amplification and sequencing primers used for deep sequencing

Exon	Product length	Amplification primers (5′-3′)	Sequencing primers (5′-3′)
2	265 bp	F: GACACAACAAAACACTCAAC	GACACAACAAAACACTCAAC
		R: TCATGCTTAGAAAACTTTGA	
3	230 bp	F: GACAGAACAGTAAAGATTCC	TGAAAGTACCATTATCACCTTTTACG
		R: GGATTGCCTTTCTTCAGTGG	
4	371 bp	F: GCCAAACAACAACCCTGGCG	TATTTAGGTTTCTTACGCAC
		R: ACAAGCTTAAGTTTCCCTCC	
5	297 bp	F: TGGACAATATATCCACCTACAGAGG	ACAGAGGCCCACAGTATTAC
		R: GATTTCAGAGAAACATATGGAGTCA	
6	450 bp	F: GCAGCTAAGCAGTGGACTAT	CACAGCCAGAAAATAGCAAT
		R: AAGATGTTAGACATGCTACT	
7	246 bp	F: TGACAATTCTTTTTCTAGACATCA	GATCTGTTTCAGAAGAGTGGA
		R: CTTTCAGTGCTCAACATCCAA	
8	301 bp	F: TCCCATAAGAGCAGAGAAGA	ATGATTTGGGTCTGGTCAGG
		R: ATTCCTCCCCTTTCTCCACA	
9	297 bp	F: GCCTCCTGTTGCAGGTGTTA	TTCAAAGTGAGTTAAATACGAAA
		R: CTGAGTCAGGAGGGAAGCAA	
10	298 bp	F: TTTTGCTTCCCTCCTGACTC	TGGACATATTTTGTGCATTG
		R: TCGCCTTTTCTTTATTGAATCTG	
11	298 bp	F: CAAGAGCATGCACATTGGATA	TTCGTATAAATGCAAGTACA
		R: TGACATTCAATTTTCTACGATTCA	

Supplementary Table S4: Primers used for quantitative real-time PCR

Gene	Primer sequence 5′-3′	Product length
RASSF6	F: ATGGCAAACTAATTGTTGAAGG	260 bp
	R: CAGGGTGTTGCTGTGATAAGATAA	
GAPDH	F: TGGAAATCCCATCACCATCT	86 bp
	R: TGGACTCCACGACGTACTCA	
